# Postprandial Glucose Spikes, an Important Contributor to Cardiovascular Disease in Diabetes?

**DOI:** 10.3389/fcvm.2020.570553

**Published:** 2020-09-18

**Authors:** Nordin M. J. Hanssen, Michael J. Kraakman, Michelle C. Flynn, Prabhakara R. Nagareddy, Casper G. Schalkwijk, Andrew J. Murphy

**Affiliations:** ^1^Diabetes Centre, Amsterdam University Medical Centre, Amsterdam, Netherlands; ^2^Department of Internal Medicine, CARIM, School of Cardiovascular Diseases, Maastricht University, Maastricht, Netherlands; ^3^Haematopoiesis and Leukocyte Biology, Baker Heart and Diabetes Institute, Melbourne, VIC, Australia; ^4^Division of Cardiac Surgery, Department of Surgery, Ohio State University, Columbus, OH, United States; ^5^Department of Immunology, Monash University, Melbourne, VIC, Australia

**Keywords:** diabete, hyperglyacemia, inflammation, RAGE (receptor for advanced glycation end products), hematopoeis

## Abstract

Clinical trials investigating whether glucose lowering treatment reduces the risk of CVD in diabetes have thus far yielded mixed results. However, this doesn't rule out the possibility of hyperglycemia playing a major causal role in promoting CVD or elevating CVD risk. In fact, lowering glucose appears to promote some beneficial long-term effects, and continuous glucose monitoring devices have revealed that postprandial spikes of hyperglycemia occur frequently, and may be an important determinant of CVD risk. It is proposed that these short, intermittent bursts of hyperglycemia may have detrimental effects on several organ systems including the vasculature and the hematopoietic system collectively contributing to the state of elevated CVD risk in diabetes. In this review, we summarize the potential mechanisms through which hyperglycemic spikes may increase atherosclerosis and how new and emerging interventions may combat this.

## Introduction

Although the pathophysiological mechanisms through which individuals develop type 1 and type 2 diabetes are different, both conditions are characterized by elevated blood glucose levels and share a similar elevated risk of cardiovascular mortality (1). Despite the fact that benefits from intensive glucose-lowering treatment were obvious in reducing microvascular complications in initial trials such as the Diabetes Control and Complications Trial (DCCT) (type 1 diabetes), the Action to Control Cardiovascular Risk in Diabetes (ACCORD) and Action in Diabetes and Vascular Disease: Preterax and Diamicron MR Controlled Evaluation (ADVANCE) trials (type 2 diabetes) showed no obvious benefit from glucose lowering treatment in the short term (2). Even though new glucose-lowering treatments, such as glucagon-like peptide 1 (GLP1) agonists and sodium glucose receptor 2 uptake inhibitors (SGLT2i) reduce major adverse cardiovascular events (MACE), it is already evident that most of the therapeutic benefit is achieved independently of their reduction in HbA1c (3). Therefore, the extent to which a reduction of hyperglycemia in diabetes directly reduces cardiovascular disease (CVD) remains controversial. Although others have found a modest reduction of coronary heart disease by glucose-lowering treatment in large meta-analyses (4, 5), the effects are somewhat disappointing with no reduction of all-cause mortality being identified. The reasons for this are not completely understood, but it is thought that benefits of glucose-lowering treatments may be partly counterweighed by an increased occurrence of severe hypoglycemic episodes associated with intensive insulin therapy. In reality, controlling traditional CVD risk factors including blood pressure and plasma lipids still remains the most successful strategy to reduce CVD mortality in diabetes (6). However, statins are less effective in people with diabetes, even if cholesterol levels are lowered equally (7), suggesting there is still a large unmet medical need for optimized cardiovascular risk-management in diabetes. The phenomenon that diabetes is characterized by high glucose levels and an increased risk of CVD, but glucose lowering treatment fails to effectively reduce this risk, is often referred to as the glucose paradox (8).

**Figure 1:**
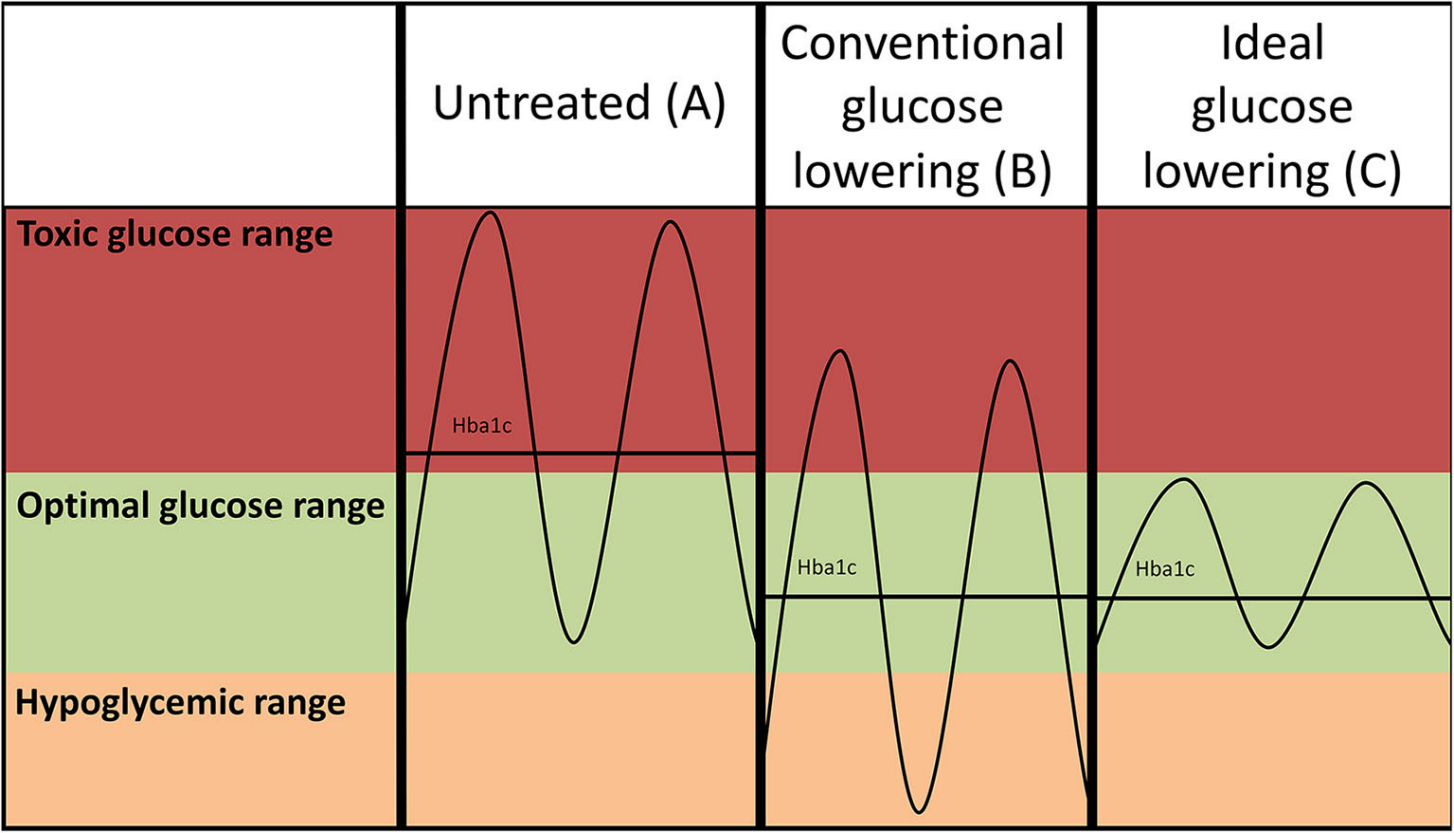
Hypothetical glucose curves of an untreated patient with diabetes **(A)** and after conventional intensive glucose lowering (insulin-based) treatment regimens **(B)**. Lowering of average glucose values may come at the expense of increased risk of hypoglycemia as the amplitude of the glucose excursions is not suppressed. Ideal glucose lowering strategies **(C)** selectively decrease postprandial glucose spikes, but do not increase risk of hypoglycemia. Please note that such a strategy reduces the amplitude of the glucose excursions, but may not necessarily further reduce HbA1c as the average glucose levels are not further reduced.

The fact that glucose lowering treatment in people with diabetes has not convincingly reduced the high risk of CVD does not rule out the possibility that high glucose levels are causally involved in the development of CVD. Studies investigating the long-term benefits of glucose lowering treatment have revealed a modest benefit of glucose-lowering treatment in both type 1 (9) and type 2 diabetes (10), and suggest that prolonged reductions are required to achieve this benefit. Epidemiological studies have also suggested that postprandial spikes of high glucose levels may be a more robust determinant of CVD risk than average glucose levels (11–15). These episodes of high glucose levels increase oxidative stress, which in turn has several detrimental downstream effects, activating immune cells, and keeping the vasculature in a persistent state of elevated risk of cardiovascular events (Figure 1). To further support this hypothesis, postprandial blood glucose (PBG) levels are more predictive for CVD than HbA1c levels. Even in people without diabetes, PBG levels independently predict CVD in the non-diabetic glucose range. If these individuals were grouped into the lowest (69–107 mg dL^−1^) vs. the highest (150–194 mg dL^−1^) PBG, there was a 27% increased risk of CVD in those that had poorer PBG control. Interestingly, these studies suggest that this association is a continuum for PBG, while for fasting plasma glucose levels there seemed to be a threshold effect at 100 mg/dL (5.6 mmol/L) (16). Along with the amplitude of the glucose spike, the duration outside of the “normal” range is also likely to be important. Recently it was shown that people with pre-diabetes spent equal amounts of time (~50% of the day) as individuals with type 2 diabetes outside of the optimal glucose range (17). Therefore, we suggest that people with diabetes (both type 1 and type 2), glucose-lowering treatment strategies directed at increasing the time in the desired glucose range may be more effective in reducing CVD in diabetes.

In this review, we will summarize the findings that lead us to this hypothesis, discussing human studies as well as mouse models of transient hyperglycemia (i.e., akin to exploring the glucose effects of PBG spikes). We propose that expansion of myeloid cells plays a central role in this process, and we will discuss potential mechanisms, which are initiated when glucose reaches toxic concentrations that may contribute to our understanding of the glucose paradox. These include but are by no means limited to; epigenetic memory, protein modifications [advanced glycation endproducts (AGEs)], and the pattern recognition receptor for AGEs (RAGE).

## Importance of Continuity to Glycemic Control: Postprandial Glucose Concentrations May Cause the Most Harm in Diabetes

Elevation of fasting and PBG levels in diabetes are determined by distinct mechanisms of defective insulin secretion and/or signaling (18), and may occur independently of each other. Generally, upon food consumption, the incretins, glucose-dependent insulinotropic peptide (GIP), and glucagon-like peptide (GLP-1) are released by intestinal L cells to suppress glucagon release and stimulate insulin secretion. It has been appreciated for decades that the incretins are responsible for 50–70% of the insulin release following an oral glucose challenge, while this dramatically drops to ~20% in people with type 2 diabetes (19). Thus, controlling PBG is more challenging in these people. Controlling carbohydrate intake, food composition, and promoting physical activity to lower PBG in people with diabetes is important, along with interventional care (20, 21).

The association between fasting plasma glucose and risk of CVD is not linear (6), suggesting that there is a certain threshold above which glucose is less tolerated and becomes toxic. Previous studies have shown that glucose levels after an oral glucose tolerance test (OGTT) are more strongly associated with carotid intima media thickness (cIMT), a marker of atherosclerosis, rather than fasting plasma glucose or HbA1c (22). In fact, post OGTT glucose levels were also more strongly associated with cardiovascular mortality than HbA1c (23). Moreover, pharmacological interventions with glyburide or repaglinide, that reduce postprandial hyperglycemia, promote regression of cIMT, suggesting an improvement in atherosclerotic CVD burden (24). Therefore, diabetic individuals predisposed to experiencing hyperglycemic spikes may be at increased risk of developing CVD.

Unfortunately, research to establish the causal effect of hyperglycemia on CVD, independent of other risk factors associated with diabetes, has been hampered by the lack of appropriate animal models (25). Most experimental models of diabetes are complicated by concomitant changes in the lipid spectrum (generally elevated VLDL/LDL and triglycerides), which makes interpretations about the specific effects of high glucose *per se* on cardiovascular disease particularly difficult to conclude. For example, the streptozotocin (STZ)-induced model of type 1 diabetes diabetes consistently accelerates atherosclerosis and vascular inflammation in the *Apoe*^−/−^ mouse. Importantly however, diabetes in this model is characterized by concomitant increases in lipid levels, when a fed chow (26) or a high fat/low cholesterol diet (27), potentially confounding any effects of high glucose alone. While these studies are important in describing the role of hyperglycemia, the mechanisms contributing to accelerated atherogenesis by hyperglycemia, independent of cholesterol, remain unclear.

However, there are some models that allow the investigation of the effects of hyperglycemia on atherogenesis without the confounding effects of hypercholesterolemia. In a model of viral-induced type 1 diabetes in *Ldlr*^−/−^ mice fed a cholesterol-free diet, Renard et al. (28) reported that hyperglycemia, independent of changes in plasma cholesterol, increased atherosclerotic lesions. This finding was also observed in another model where *Ldlr*^+/−^ mice were employed. Rendering these mice diabetic with STZ and feeding a cholesterol/cholic acid-containing diet produced similar cholesterol levels between the diabetic and non-diabetic mice and still resulted in accelerated atherosclerotic lesion formation (29). Conversely, while the induction of diabetes in *Ldlr/Apoa-I* double knockout mice fed a cholesterol-enriched diet also had no effect on lipid levels, these mice did not develop larger atherosclerotic lesions (30). However, it must be noted that the deletion of *Apoa-I* (i.e., no HDL), could have altered the lesions in the non-diabetic group.

**Figure 2:**
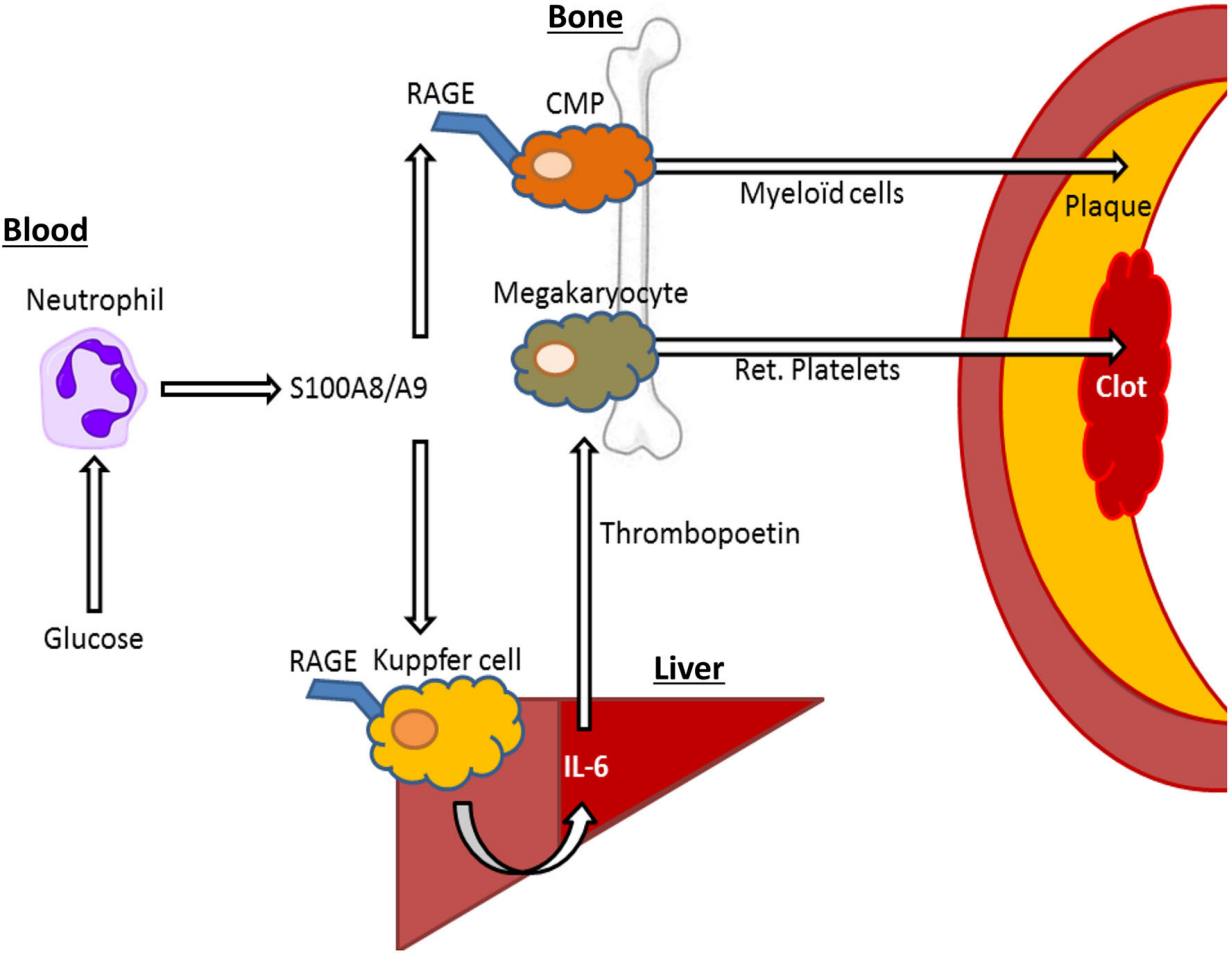
S100A8/A9—RAGE interactions on Kupffer cells and common myeloid progenitor (CMP) cells may drive elevated cardiovascular risk by hyperglycemia. In response to hyperglycemia, neutrophils release S100A8/A9, interacting with RAGE in Kupffer cells in the liver, leading to IL-6 mediated thrombopoietin release by hepatocytes that in turn leads to increased reticulated (ret.) platelet production, that contribute to atherothrombosis. Furthermore, S100A8/A9 interacts with RAGE on CMPs, leading to increased differentiation into neutrophils and monocytes (myeloid cells), which migrate to the plaque promoting plaque growth and inhibiting plaque regression.

We also designed a model to isolate the effects of hyperglycemia from lipids, but in a clinically relevant model of atherosclerotic lesion regression. This was done by establishing lesions in *Ldlr*^−/−^ mice by feeding them a modified Western Type Diet (WTD; 0.15% cholesterol as opposed to 0.2%) for 16 weeks and then switching the mice to a chow diet to lower plasma cholesterol and induce lesion regression. A group of mice were made diabetic with STZ in this period and importantly, plasma cholesterol levels were reduced to similar levels compare to control mice. In this study we also treated a group of diabetic mice with a sodium glucose cotransporter 2 inhibitor (SGLT2i), isolating the effects of glucose. We made the discovery that hyperglycemia impaired atherosclerotic lesion regression by promoting enhanced monocyte production from the bone marrow, causing their persistent entry into the atherosclerotic plaque (31) (Figure 2). This suggests that inflammatory changes in at least type 1 diabetes models are largely mediated by hyperglycemia, and not merely by changes in insulin or lipid levels.

In addition to the above-mentioned studies reporting into long-term effects of high glucose on atherosclerosis, several *in vivo* models to assess the acute effects of high glucose on the vasculature have also been developed. Firstly, increasing glucose levels up to 6 h in mice during a hyperglycemic clamp induced the sustained expression of inflammatory genes in the vasculature even after 6 days of normoglycemia (32). Interestingly, these effects were recapitulated by merely administering a series of glucose boluses in non-diabetic mice (33) suggesting that the dynamics of the glucose excursion following glucose administration may an important predictor of cardio-metabolic health. In fact, we recently showed that the repeated administration of such hyperglycemic spikes robustly increases atherosclerosis and myeloid cell expansion in otherwise normoglycemic *Apoe*^−/−^ mice, while their lipid profile and HbA1c remained unaltered (34).

In conclusion, experimental studies have supplied evidence that glucose contributes to increased atherosclerosis in diabetes independently of changes in lipids or insulin. However, the relative contribution of glucose to atherogenesis seems to differ greatly between mouse models and the use of atherosclerosis-prone diets. Profound hyperlipidemia in some models may “mask” any contributions by high glucose, and this phenomenon remains poorly understood. As lipid metabolism differs greatly between mice and humans (35), the extent to which these findings translate to humans is unknown. Future studies to address this issue are clearly warranted.

## Which Tissues are Sensitive to High Glucose With Relevance to CVD?

Atherosclerosis is a systemic vascular disease, in which a complicated interplay between the pre-existing cells of the vasculature and recruited inflammatory cells occurs, forming an advanced atherosclerotic lesion. Experimental studies have identified several cell types including endothelial cells and immune cells that are especially sensitive to high glucose levels.

The endothelium appears particularly sensitive to changes in glucose, likely due to its constant exposure to blood glucose fluctuations. Despite expressing the insulin receptor, endothelial cells are unable to restrict the amount of glucose they uptake, as the glucose transporter 1 (GLUT-1) is not down-regulated in response to high glucose (36). Furthermore, more recent findings indicate that increases in circulating myeloid cells, derived from bone marrow progenitors, are directly influenced by changes in blood glucose and are not merely due to secondary responses to vascular injury. For example, in response to high blood glucose levels, neutrophils secrete the damage associated molecular pattern (DAMP) molecules such as S100A8/A9 which interact with RAGE on myeloid progenitor cells in the bone marrow to promote monocytosis and impair lesion regression (31) (Figure 2). Exposing neutrophils to high levels of glucose results in their activation and release of S100A8/A9 via increased glycolytic flux and generation of reactive oxygen species (34). Diabetes, through neutrophil-released S100A8/A9, also causes liver inflammation (37). This occurs through interaction with RAGE on Kupffer cells, in turn promoting the production of IL-6, which stimulates thrombopoietin (TPO) production in the liver. We found that the increased production of TPO promoted platelet production, which appeared to be responsible for reticulated thrombocytosis, commonly seen in people with diabetes (37) (Figure 2). These immature platelets are often more reactive and inflammatory that their mature counterparts and can accelerate atherosclerosis, as we revealed in diabetes and through genetic models of enhanced production (37–39). Thus, we suggest that neutrophils are an innate sensor of hyperglycemia to promote glucose-induced inflammation.

## Transient High Glucose Exposure May Contribute to Sustained Risk of CVD

In an attempt to explain the lack of efficacy and/or delayed response in glucose lowering treatment in reducing CVD risk in diabetes, several authors have postulated that high glucose induces a metabolic memory (or causes a so-called legacy effect). According to this hypothesis, a high glucose induced phenotype is “remembered” by the vasculature, which in an individual with diabetes develops into a persistently increased risk of CVD, even when blood glucose is lowered (Figure 1B). Support for a metabolic memory to high glucose arose from the findings from the long-term follow-up of the DCCT. Despite the fact that a difference in HbA1c was no longer detectable between the intensive glucose lowering and control group after the initial trial period, the individuals from the intensive glucose-lowering treatment group did display a reduced risk of CVD at long-term follow-up relative to the control group (9). Furthermore, STZ treated *Apoe*^−/−^ mice in which their diabetes regressed and thus were only transiently hyperglycemic, displayed equal plaque development compared to STZ injected *Apoe*^−/−^ mice that remained diabetic (40). Although the mechanisms that contribute to metabolic memory are still far from clear, the endothelium seems particularly susceptible to metabolic memory. For example, transient exposure of cultured endothelial cells to high glucose was sufficient to induce a persistent increase in p65 expression, a major subunit of the NF-κB complex, (32). In fact, when non-diabetic mice were subjected to a hyperglycemic clamp, the increased p65 expression in endothelial cells isolated from the aorta persisted for up to 1 week. This memory appears to be associated with the accumulation of reactive oxygen species (ROS), as ROS production continued for at least 1 week after normalization of glucose and was paralleled by an increase in inflammatory gene expression, which could be reduced with a ROS inhibiting agent (41). Whether glucose imparts significant epigenetic modification in hematopoietic cells (stem or mature) that directly influence atherosclerosis is yet to be fully understood, but could also be an important mechanism in which transient high glucose levels contribute to CVD.

Recently, we explored PBG spikes using a model termed “transient intermittent hyperglycemia” in mice, by injecting glucose four times in a day spaced 2 h apart (34). We discovered that exposure to these PBG spikes 1 day per week accelerated atherogenesis, which was driven by enhanced myelopoiesis. The transient rises in blood glucose activated circulating neutrophils through glycolysis and oxidative stress, which support previous hypotheses.

## AGEs

AGEs are irreversible, long-term sugar modifications of proteins. Therefore, it is likely that once formed in the vasculature during hyperglycemia, they lead to a long-term disruption of cellular function even after blood glucose has been normalized. The accumulation of AGEs, first described as consequence of heating it (42), was considered mostly a slow and passive process *in vivo* (43), especially for AGEs such as pentosidine or glucosepane which have been linked to crosslinking of vascular collagen, leading to vascular remodeling, arterial stiffness and which, in turn, may increase cardiovascular risk (44). Indeed, in line with this concept, Monnier et al. (45) showed lower AGE levels in skin biopsies of DCCT subjects that received intensive glucose-lowering treatment.

Furthermore, rapid AGE formation from highly reactive intermediates of glycolysis is now considered the major mechanism of AGE formation *in vivo* (46). Methylglyoxal (MGO) has been identified as the major precursor for AGEs (47). MGO is a byproduct of glycolysis, and in conditions of intracellular hyperglycemia leads to a rapid increase of AGEs (36). MGO is increased in diabetes, closely tracks with PBG levels (48) and is associated with CVD in type 1 (49) and type 2 diabetes (50). Prevention of MGO and AGE accumulation by overexpressing glyoxalase 1 (GLO1) (51), the major enzyme to detoxify MGO, prevented endothelial dysfunction (52) and ROS formation (51) in STZ treated rats. MGO has not only been linked to the modification of cytosolic proteins, but also has been shown to directly modify and damage DNA (53). In fact, a link between accumulation of MGO and development of a epigenetic memory has been made by El-Osta et al. Overexpression of GLO1 prevented the persistent increase of p65 expression in endothelial cells following transient hyperglycemia, while GLO1 knockdown mice, displayed increased p65 expression in their endothelial cells even when normoglycemic (32). This is perhaps not surprising, given that MGO accumulation is closely linked to formation of ROS (52). The lowering of AGEs has been proposed as a treatment for diabetic CVD, as AGE inhibiting compounds, such as alagebrium, and pyridoxamine have been shown to reduce atherosclerosis in diabetic *Apoe*^−/−^ mice (26). However, it is important to note that AGE levels within the atherosclerotic lesions of *Ldlr*^−/−^ mice failed to correlate with lesion size (54), thus it is still debated where AGEs impart their atherogenic effects.

While elevated AGEs (specifically the AGE moieties N^ε^-(carboxyethyl) lysine (CML) and pentosidine) are observed in people (55) and animals with type 1 diabetes (51), plasma AGE [CML, pentosidine and N^ε^-(carboxyethyl) lysine (CEL)] levels are not increased in individuals with type 2 diabetes (56). This discrepancy may be explained by the fact that formation of AGEs is a complex and heterogeneous process, and AGEs may also be formed as a result of glucose-independent processes such as lipid-oxidation and other types of ROS (57). Furthermore, AGE levels (of CML and 5-hydro-5-methylimidazolone (MG-H1) are strongly increased in unstable plaques, but these plaque AGE levels were not associated with plasma glucose levels, and were not higher in individuals with type 2 diabetes (58). Together, these findings indicate that AGE levels are not determined by hyperglycemia alone. In fact, plaque CML and MG-H1 levels may be produced in response to plaque inflammation and hypoxia, rather than hyperglycemia (58). Nonetheless, plasma levels of CML and CEL were strongly associated with incident CVD in both individuals with type 1 diabetes (59), and type 2 diabetes (60), although in type 2 diabetes this association became much stronger for CML after adjustment for BMI. This finding is likely explained by the fact that obese adipose tissue traps certain circulating AGEs (CML in particular) in a RAGE-dependent manner, reducing their plasma levels (61). Interestingly, although crosslinking AGE pentosidine was associated with CVD in type 1 diabetes, this association was not present in type 2 diabetes (60, 62). Taken together, these results also underline that type 1 and type 2 diabetes are metabolically distinct diseases, and therefore yield different patterns of AGEs.

Few clinical studies have been performed to investigate whether AGE-lowering compounds are suitable for use in humans. Unfortunately, aminoguanidine, the best known AGE-inhibitor induced glomerulonephritis in a small subset of subjects at higher dosages (63), and its use in clinical studies has therefore been discontinued. Other well-known AGE inhibiting compounds, such as alagebrium, have been used in small clinical trials with mixed results (64). It remains to be tested if these or any other AGE-lowering compounds will reduce CVD endpoints in individuals with either type 1 or type 2 diabetes (64), but based on these initial studies, efforts should be made to develop new AGE-lowering compounds with stronger AGE inhibition, but fewer non-glycation directed properties, yielding a more favorable toxicity profile.

## Rage

A number of studies have shown that RAGE plays a pivotal role in the development of atherosclerotic lesions in diabetes, as deletion of RAGE almost completely reduces the extent of atherosclerosis in diabetic *Apoe*^−/−^ mice (65). Since RAGE deletion also reduces atherosclerosis in non-diabetic mice, its protective effects on atherosclerosis cannot be fully derived from prevention of high glucose dependent effects. We showed that glucose induced monocytosis, a major determinant of plaque growth, was almost completely dependent on hyperglycemia-dependent RAGE signaling in the bone marrow (31). However, RAGE did not mediate increased monocytosis in a model of obesity and only mild hyperglycemia (66), suggesting a threshold above which glucose levels induce RAGE signaling. RAGE may thereby mediate a sustained inflammatory response induced by hyperglycemic spikes by releasing increased amounts of monocytes in the circulation. Further complexity for the involvement of RAGE in glucose mediated vascular damage was discerned as hematopoietic or tissue (non-hematopoietic) deletion of RAGE equally inhibited atherosclerosis in diabetic *Apoe*^−/−^ mice (67). Perhaps the link is that RAGE is associated with sustained NF-κB activation (68), and as such, a major link between hyperglycemia, ROS, and inflammation.

There is also compelling evidence that RAGE is associated with diabetes and CVD in humans. RAGE expression is increased in plaques of the carotid artery of individuals with type 2 diabetes (69), which can be lowered with statin treatment (70). Although the exact consequence of increased RAGE expression in human atheroma is not clear, it does not seem to influence or predict plaque vulnerability directly, as the expression of RAGE does not differ between stable or ruptured plaque segments (58). However, RAGE has been shown to co-localize with MMP9, a major marker for plaque rupture (70).

In addition to its role as the receptor for AGEs, RAGE is also a well-recognized pattern recognition receptor that can interact with various inflammatory molecules, such as S100A8/A9, S100B, and HMGB1. It is difficult to discern which ligand for RAGE plays the most important role in the development of CVD, as S100A8/A9, S100B, and HMGB1 as well as the AGEs have all been shown to increase due to high glucose (71), and are associated with CVD (58, 62, 72). S100A8/A9 appear to be the important biological ligands of RAGE, at least in driving monocyte and neutrophil production from the bone marrow. Transplantation of *S100a9*^−/−^ bone marrow into WT mice that were subsequently made diabetic with STZ were protected from hyperglycemia-induced leukocytosis (31). Additionally, depletion of neutrophils (the main source of S100A8/A9) in diabetic mice normalized S100A8/A9 levels and normalized circulating blood monocyte levels but had no effect on the expression of RAGE on the bone marrow common myeloid progenitors CMPs (31). This suggests that at least in respect to monocyte production, other RAGE ligands (i.e., AGEs and HMGB1) are insufficient in stimulating RAGE signaling in the CMPs to produce monocytes. Further, when we isolated neutrophils from mice with diabetes or diabetes + SGLT2i we observed that lowering blood glucose normalized the expression of *S100a8* and *S100a9*, without impacting *Hmgb1* expression, providing more evidence for the importance of S100A8/A9 compared to HMGB1 (31). In line with our hypothesis that glucose and PBG spikes drive atherogenesis in mice, these same pathways were initiated in mice exposed to transient intermittent hyperglycemia (34).

## Approaches to Reduce Postprandial Hyperglycemia and Reduce CVD

Traditionally, intensive insulin-based regimens have been hampered by concomitant increase of hypoglycemia (Figures 1A,B), and intensification of glucose-control with insulin use has even been linked to an increase of all-cause mortality in vulnerable individuals with type 2 diabetes (73). Higher frequencies of hypoglycemia are associated with a higher risk of CVD (74). The mechanism through which hypoglycemia increases risk of CVD is not completely understood, but profound sympathetic activation in response to low glucose, leading to strong hemodynamic changes, is presumed to play a large role (75). New approaches to lower glucose have been developed to reduce postprandial hyperglycemia with a lower risk of hypoglycemia events. The underlying concept would be to lower glucose excursions (Figure 1C) rather than lowering average glucose which comes at the expense of increased risk of hypoglycemia (Figure 1B). A novel approach to refine insulin use is the development of a bionic pancreas (76). This allows for continuous glucose monitoring such that insulin as well as glucagon release are automated, yielding much more precise glucose control. Furthermore, as mentioned below SGLT2i and GLP1 agonists, which effectively lower hyperglycemia, also appear to avoid hypoglycemic events. Since their striking cardiovascular effects have mainly been addressed in cardiovascular safety studies, at short follow-up times, it is actually possible their true benefits will be even greater at longer duration of use, as benefits of glucose-lowering treatments take a long time to cause clinically meaningful results.

### Sodium Glucose Co-transporter 2 Inhibitors

Arguably one of the most important drug developments over the past decade to treat people with diabetes is the SGLT2i's. SGLT2i's empagliflozin, dapagliflozin, and canagliflozin, have been linked to striking reductions in heart failure and chronic kidney disease in their land-mark cardiovascular safety studies (EMPA-REG, DECLARE, CANVAS). Their effect on HbA1c however, is overall modest, and their effect on MACE is most likely mediated by glucose-independent, mainly hemodynamic, mechanisms (77). Although SGLT2i do not carry an inherent risk of hypoglycemia, their use has been linked to increased risk of a (normoglycemic) ketoacidosis, warranting careful use of these compounds in type 1 diabetes. Nonetheless, continuous glucose monitoring studies have revealed interesting effects of these compounds on glucose variability.

The effect of SGLT2i on hyperglycemic excursions seems to be largely influenced by the context in which they are given. For instance, empagliflozin monotherapy was found not to reduce postprandial glucose excursions or 24-h glucose variability (78), while when added to insulin therapy empagliflozin significantly lowered glucose excursions in type 2 and even in type 1 diabetes. Sotagliflozin, an inhibitor of both the SGLT1 and SGLT2 receptor, has also been shown to substantially decrease the number of hyperglycemic episodes in individuals with type 1 diabetes (79). These beneficial effects on glucose variability are likely achieved in large part due to decreased insulin dosages leading to less pronounced glucose peaks, and less offshoot effects by hypoglycemia, since SGLT2i mainly increase renal glucose excretion.

### GLP-1 Agonists

GLP-1 agonists (e.g., liraglutide, semaglutide, exenatide, etc) lower HbA1c by 1–2% points compared to usual care plus placebo and reduce cardiovascular disease (80, 81). The profile of their beneficial cardiovascular effect differs considerably from SGLT2i's, with less pronounced reductions in heart failure and more overall reduction in CVD. This likely reflects the distinct mechanism of action of GLP-1 agonists compared to SGLT2i's. Given the pleiotropic effects of GLP-1 agonists, the precise mechanisms of action leading to cardiovascular risk reduction is unclear. Interestingly, the FLAT-SUGAR Trial has shown that exenatide on top of long-acting insulin reduced glucose excursions more than a four daily insulin regimen (82). Thus, reductions in glucose excursions may be, at least in part, an important cardio-protective mechanism offered by GLP-1 agonists and warrants further investigation.

### Designer Cytokines

Over the past few decades, an appreciation of the interaction between cytokines and metabolic regulation has evolved. While most cytokines relay important signals around the body during times of inflammation, some cytokines also serve important basal roles. Once such cytokine is IL-6, known to promote inflammation under many conditions, but also to be increased during bouts of exercise with positive effects on metabolism via GLP-1 (83). A recent study from the Febbraio group took the novel approach of generating a cytokine-like molecule that combined IL-6 and the leukemia inhibitor factor receptor (LIFR) binding domain of ciliary neurotrophic factor (CNTF) (84). This unique designer cytokine, termed IC7Fc, was able to retain the positive metabolic, without the inflammatory effects of IL-6, while also preserving CNTF's effects on satiety. This was achieved by the molecule preferentially docking to the gp130 cytokine signaling receptor with either the LIFR or the IL-6R. When administered to mouse models of obesity and diabetes, IC7Fc was able to prevent weight gain and in turn improve glucose tolerance and hyperglycemia, along with protecting the liver from steatosis (84). Positive effects were also seen in the musculoskeletal system. However, no cardiovascular outcomes were reported which is critical in the development and approval of therapies against metabolic diseases. Although, one would hypothesize that, given the strikingly positive effects on metabolism, IC7Fc may also reduce CVD. Another interesting observation that leads us to hypothesize that IC7Fc could protect against CVD was the positive effects on the bone. Extending this observation, we ponder if IC7Fc treatment would impact the hematopoietic stem cell microenvironment to retain stem cells and prevent unwanted extramedullary hematopoiesis which has been shown to directly influence atherosclerosis (85, 86). This new class of therapy in the area of cytokines, opens up other avenues where cytokines may be utilized to have positive effects on metabolism. These could include NLRP1/IL-18 axis and IL-33, both of which can have multiple roles in inflammation, but potent anti-obesity effects (87). Whether targeting any of these pathways could limit glycemic variation and ultimately reduce CV events is an exciting prospect and remains to be explored.

## Conclusion

Evidence suggests some reduction in CVD risk by long-term glucose lowering treatments in people with diabetes. This effect however, seems limited to certain specific subpopulations, and is minor in type 2 diabetes with a complicated risk spectrum at best, with some studies even reporting harmful effects due mostly to hypoglycemia (74). Nonetheless, epidemiological research now suggests that postprandial high glucose “spikes” as opposed to high average glucose levels are a more important determinant in CVD development in diabetes. Therefore, we argue that new glucose lowering strategies should be more directed against the reduction of postprandial spikes, than of HbA1c and/or average glucose, as such a strategy should avoid concomitant episodes of hypoglycemia. The extent to which HbA1c captures transient hyperglycemic episodes seems to be highly dependent on the type of diabetes, and degree of overall glycemic control.

Based on the transient hyperglycemia experiments in mice, we speculate that hyperglycemic spikes may be sufficient to sustain an individual's increased risk of CVD. Importantly, pre-clinical studies have also established that hyperglycemia plays a causal role in the development of CVD in diabetes and even impairs the resolution of lesions in the setting of cholesterol lowering. This can be reversed by the administration of a SGLT2i's. If this translates into clinical outcomes with SGLT2i's and other new interventions, without the risk of hypoglycemia, this would suggest that such compounds would be attractive combat CVD in diabetes. Whether the SGLT2i can sufficiently reduce hyperglycemic spikes and prevent its consequences in humans remains to be determined. In humans, their use has been showed to significantly reduce hyperglycemia (88), and therefore, clinical trials evaluating their effect on cardiovascular risk are eagerly awaited. The limited risk-reducing effects from insulin treatment have been attributable to the low compliance issues caused by subsequent weight gain and episodes of hypoglycemia. Hopefully, the development of the bionic pancreas will improve benefits of intensive insulin use. Whether the bionic pancreas will have any place in the management of advanced type 2 diabetes remains to be determined.

Compounds erasing epigenetic marks, inhibiting formation of AGEs and signaling of the RAGE receptor may provide promising therapeutic targets for treating the consequences of hyperglycemic spikes in diabetes. The molecular mechanisms on which these compounds operate are closely linked and may form a positive feedback loop through formation of ROS. Now, large clinical trials are needed to evaluate whether these new compounds actually reduce the risk of cardiovascular disease in humans with both type 1 and type 2 diabetes, bearing in mind that the results may differ in the two very different diseases. Furthermore, additional mechanisms, beyond the score of the current review, such as impaired collateral vessel formation, may play a role in the detrimental effects of hyperglycemic spikes.

## Clinical Perspective

Based on experimental studies, several strategies have been identified to reduce the risk of CVD in people with diabetes. These include reducing hyperglycemic spikes, epigenetic marks, accumulation of AGEs, and inhibiting RAGE ligands. However, we are still awaiting the evaluation of compounds intervening with these pathways in large-scale clinical trials.

## Author Contributions

All authors contribute to writing and editing the manuscript.

## Conflict of Interest

The authors declare that the research was conducted in the absence of any commercial or financial relationships that could be construed as a potential conflict of interest.

## Abbreviations

AGEs: Advanced glycation endproducts
CVD: Cardiovascular disease
CMPs: Common myeloid progenitors
cIMT: Carotid intima media thickness
HbA1c: Glycated hemoglobin
GLO1: Glyoxalase 1
HDACi: Histone-deacetylase inhibitors
RAGE: Receptor for advanced glycation endproducts
ROS: Reactive oxygen species
SGLT2i: Sodium glucose cotransporter 2 inhibitor
STZ: Streptozotocin.
